# Hitting an uncertain target

**DOI:** 10.7554/eLife.18721

**Published:** 2016-07-15

**Authors:** Veit Stuphorn

**Affiliations:** 1 Department of Psychological and Brain Sciences, Johns Hopkins University, Baltimore, United Statesveit@jhu.edu; 2 Department of Neuroscience and the Krieger Mind/Brain Institute, Johns Hopkins School of Medicine, Baltimore, United States

**Keywords:** single neurons, reaching, planning, decision making, Rhesus macaque

## Abstract

Experiments in which monkeys have to select or estimate the location of a target are revealing more about the role of the dorsal premotor cortex in decision making.

**Related research article** Dekleva BM, Ramkumar R, Wanda PA, Kording KP, Miller LE. 2016. Uncertainty leads to persistent effects on reach representations in dorsal premotor cortex. *eLife* **5**:e14316. doi: 10.7554/eLife.14316

**Image** Maps of neural activity in the dorsal premotor cortex (left) and the primary motor cortex (right)

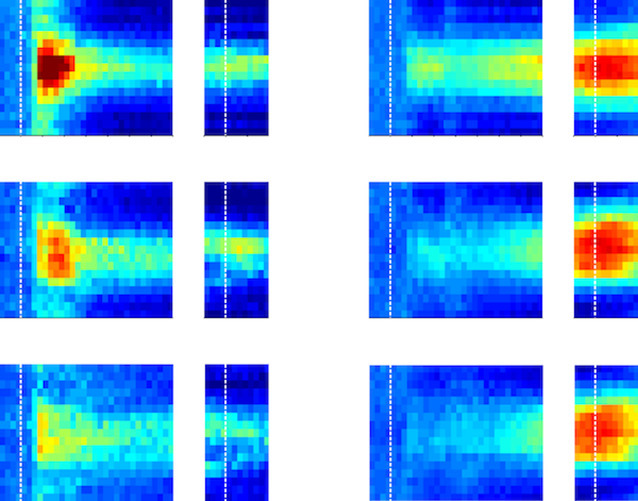

Life is full of uncertainty. Take, for example, a football player preparing to take a penalty kick. He or she can aim to kick the ball along a number of different trajectories, and the success of the kick will depend on a range of different factors, such as small differences in the execution of the kick and, most importantly, the response of the goalkeeper. The simplest way to think about the decision that the player has to make is to say that he or she has to decide between two options: aiming for the left half of the goal or aiming for the right half (Figure 1A). Most studies of the neuronal mechanisms that underlie decision-making have focused on "target selection" situations like this, where the available options are limited in number and clearly distinct from each other (Shadlen and Newsome, 2001; Romo et al., 2004; Thura and Cisek, 2014).

**Figure 1.**
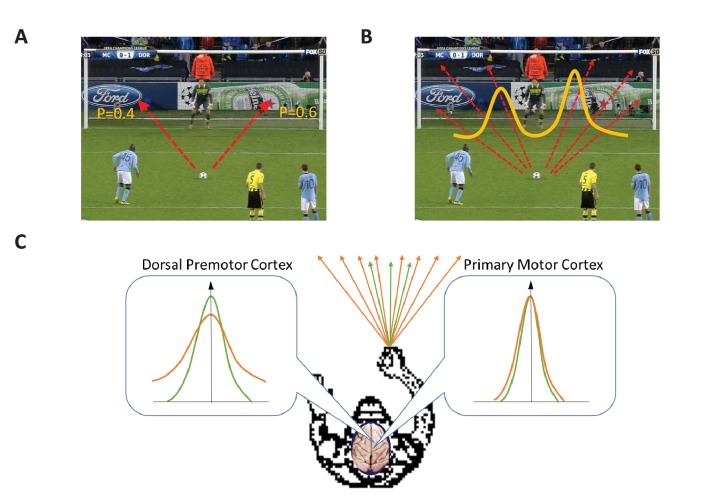
Target selection and target estimation. (**A**) In a target selection situation there is a choice between two or more, clearly distinct, options. In this example there are two options (indicated by the two red arrows), and each option is associated with a specific probability of success (indicated by the numbers). A region of the brain called the dorsal premotor cortex plays an important role in making target selection decisions (also known as categorical decisions), while the primary motor cortex is responsible for executing the decision. (**B**) In a target estimation situation there is an infinite number of options (six of which are indicated by red arrows), and the probability of success can be plotted as a distribution with two peaks (yellow line). (**C**) Dekleva et al. have performed experiments on monkeys to explore the roles played by the dorsal premotor cortex and the primary motor cortex in making target estimation decisions (also known as continuous decisions). In the experiments the monkeys had to select a specific direction from a range of possible directions on the basis of incomplete visual information. Dekleva et al. measured how the level of neural activity (y-axis) in these two regions of the brain varied as a function of the angle (x-axis) between the preferred direction of the neurons and the chosen direction; they also varied the degree of uncertainty in the visual information provided to the monkeys about the location of the target. The range of possible directions was wide (orange arrows) when the uncertainty in the visual information provided to the monkeys was high, and the range was narrow (green arrows) when the uncertainty was low. In both regions the level of neural activity was highest when the angle between the preferred direction and the chosen direction (indicated by the vertical arrow) was zero. However, high levels of neural activity were observed over a relatively wide range of angles in the dorsal premotor cortex (left inset), and this range was higher when the degree of uncertainty was higher (orange line). In the primary motor cortex, on the other hand, high levels of neural activity were only observed for a narrow range of directions around the chosen direction, independent of the degree of uncertainty in the visual information (right inset).

However, the player taking the penalty obviously has more than two options: they can, for example, aim low, which increases their chances of hitting the goal, but also gives the goalkeeper a better chance to make a save; or they can aim high to make it more difficult for the goalkeeper and themselves. Indeed, they can aim at any point in the goal. This means that the player has to decide between an infinitely large range of possible trajectories (Figure 1B). To do this, he or she has to estimate the probability of success for each possible trajectory and then select the one with the highest likelihood of success. In the language of neuroscience the player is confronted with a "target estimation" situation.

How might the brain make a decision in such a situation? Studies of primates suggest that a region of the brain called the dorsal premotor cortex plays an important role in making target selection decisions (Cisek and Kalaska, 2005), and that the primary motor cortex is responsible for executing such decisions. Now, in eLife, Lee Miller and colleagues at Northwestern University, including Brain Dekleva as first author, report the results of experiments on monkeys that shed new light on the role of these two regions in making target estimation decisions (Dekleva et al., 2016). The experiments involved simultaneously recording neural activity in the dorsal premotor cortex and the primary motor cortex as the monkeys performed a target estimation task.

Playing football would be impractical in an electrophysiological experiment so, instead, two monkeys were trained to use a device called a manipulandum to move a cursor on a screen. The experiment involved two types of trials. During the first type of trial the monkey held the cursor over a central target: then, after a random period of time, the central target disappeared and a new target appeared in one of eight locations (which were arranged in a circle around the central target). The monkey received a reward every time it moved the cursor to the correct target. Neurons in the dorsal premotor cortex (PMd neurons) and the primary motor cortex have a "preferred direction", and the Northwestern group measured the level of neural activity as a function of the angle between the preferred direction of groups of neurons and the direction selected by the monkey (Figure 1C).

During the second type of trial the location of the new target was indicated by number of small lines that were sampled from a distribution that was centered on the target location. (There were five small lines for one monkey, ten for the other). The lines supplied the monkeys with incomplete information about the target location, and Dekleva et al. were able to vary the degree of uncertainty in this information by varying the width of the distribution from which the lines were sampled. This meant that the monkeys were now performing a target estimation task.

When the degree of uncertainty in the visual information supplied to the monkeys was high they tended to move the cursor to a location that was the average of the target locations in the previous trials: this approach makes sense when relatively little information is available. The Northwestern group also observed relatively high levels of activity in the PMd neurons representing directions other than the selected direction when the uncertainty was high: this suggests that, during decision making, the dorsal premotor cortex represents all directions that might be correct, each weighted by its probability of success. Again this makes sense: when the degree of uncertainty is high, there is a higher likelihood that a direction other than the chosen one is correct, so the PMd neurons representing these other directions are more active. In contrast, in the primary motor cortex, only those neurons representing the chosen direction were active (Figure 1C).

Previous studies of target selection reported that the activity of PMd neurons representing the chosen action increased during decision making, whereas the activity of other PMd neurons decreased (Cisek and Kalaska, 2005; Thura and Cisek, 2014). This was taken as evidence that the dorsal premotor cortex plays a direct role in decision making. In contrast, by showing that the PMd neurons representing all possible actions are active throughout the planning and execution of a target estimation task, the results of the Northwestern team argue strongly against a direct role for the dorsal premotor cortex in decision making because it is the M1 neurons, but not the PMd neurons, that encode the chosen direction unambiguously.

Future experiments should compare the role of PMd neurons in both target selection and target estimation tasks. Examining situations that are intermediate between target selection and target estimation might also help to resolve the difference between the latest findings and previous work.

The latest results also shed some light on the question how probability and uncertainty are encoded in the brain. There is some evidence for an explicit representation of uncertainty in the orbitofrontal cortex of primates (O'Neill and Schultz, 2010). However, recent work in rodents indicates that other interpretations are possible (Ogawa et al., 2013), and the results of Dekleva et al. support an implicit (rather than explicit) representation (Ma et al., 2006).

By pioneering the study of an important class of real-world situations that have been neglected up to now, the work of the Northwestern group opens up a rich new field of investigation.
